# The CHRNA5 rs16969968 variant is associated with MMP-9 expression and inflammatory signaling in COPD in a West Bengal population, India

**DOI:** 10.3389/fphar.2026.1841945

**Published:** 2026-06-24

**Authors:** Himani Adhikari, Nasima Sultana, Achintya Mohan Goswami, Indranil Ganai, Himani Biswas, Amalesh Mondal, Asif Iqbal, Saibal Moitra, Sanjoy Podder

**Affiliations:** 1 Ecology and Allergology Laboratory, Department of Zoology, The University of Burdwan, Burdwan, West Bengal, India; 2 Department of Physiology, Krishnagar Govt. College, Krishnagar, West Bengal, India; 3 Department of Zoology, Lady Brabourne College, Kolkata, India; 4 Department of Physiology, Katwa College, Katwa, West Bengal, India; 5 Apollo Multispecialty Hospitals, Kolkata, West Bengal, India

**Keywords:** CHRNA5, COPD susceptibility, immunogenetics, inflammatory remodeling, *in-silico* analysis, MMP-9

## Abstract

**Background:**

Host genetic variation plays a critical role in shaping inflammatory responses to chronic environmental exposure in Chronic Obstructive Pulmonary Disease (COPD). The non-synonymous CHRNA5 polymorphism rs16969968 (G>A; D398N) has been associated with nicotine dependence; however, its immunomodulatory relevance in COPD remains insufficiently explored.

**Objectives:**

To investigate the association of the CHRNA5 rs16969968 polymorphism with COPD susceptibility and its relationship with inflammatory protease expression and cholinergic signaling pathways in a West Bengal population.

**Methods:**

We enrolled 412 COPD patients and 390 controls (41–80 years). Lung function was assessed by spirometry. Genotyping was performed using the PCR-RFLP method. CHRNA5 and MMP9 mRNA expression levels were quantified by qRT-PCR, and CHRNA5 protein expression was assessed via Western blot; BALF MMP-9 levels were measured by ELISA. Computational tools were used to predict the potential structural and functional effects of the variant.

**Results:**

The rs16969968 AA genotype was significantly enriched among COPD patients and was associated with reduced pulmonary function in smokers. Carriers of the risk genotype reported lower CHRNA5 transcript and protein expression levels along with higher MMP-9 expression compared with other genotypes. In-silico analyses also predicted that the variant D398N likely to exert subtle functional effects on α5 nicotinic acetylcholine receptor subunit.

**Conclusion:**

The findings suggest that the CHRNA5 rs16969968 variant is associated with altered cholinergic signaling and increased MMP-9 expression in COPD. These observations support a potential role for CHRNA5-related inflammatory pathways in COPD susceptibility and tissue remodeling. However, the observed associations require further functional and mechanistic validation.

## 1 Introduction

Chronic Obstructive Pulmonary Disease (COPD) is a progressive inflammatory disorder of the lower respiratory tract characterized by persistent airflow limitation and structural remodeling of the airways and alveolar compartments (Sultana et al., 2025). Beyond its clinical manifestations, COPD is increasingly recognized as a heterogeneous disease driven by dysregulated innate and adaptive immune responses to chronic environmental exposures. Large COPD cohorts, including COPDGene, SPIROMICS and ECLIPSE have established substantial clinical, physiological, imaging and molecular heterogeneity in COPD, demonstrating marked variability in emphysema burden, airway remodeling, inflammatory profiles, exacerbation frequency and disease progression (Regan et al., 2011; Couper et al., 2014; Vestbo et al., 2008). Recent multidimensional phenotyping studies, including the work of Fangal et al. (2024), further demonstrated that COPD in smokers exhibits distinct physiological, imaging and transcriptomic characteristics compared with asthma and asthma-COPD overlap, including greater emphysema, gas trapping, functional small-airway disease and COPD-specific inflammatory programs (Fangal et al., 2024). These observations highlight the importance of identifying molecular pathways and genetic determinants that contribute specifically to COPD susceptibility and progression. Host genetic determinants that influence inflammatory signaling pathways are therefore considered critical contributors to inter-individual variability in COPD susceptibility and disease severity (Qi et al., 2024). Accumulating evidence indicates that single nucleotide polymorphisms (SNPs) within genes regulating inflammatory and cholinergic signaling pathways contribute to variability in COPD risk. Among these, variants within the nicotinic acetylcholine receptor (nAChR) gene cluster on chromosome 15q25.1 have attracted particular attention (Pillai et al., 2010). The CHRNA5 gene encodes the α5 subunit of nAChRs, ligand-gated ion channels expressed not only in neuronal tissues but also in non-neuronal pulmonary cells, including airway epithelial and immune cells (Borkar et al., 2024). These receptors participate in the cholinergic anti-inflammatory pathway, modulating intracellular calcium flux, cytokine production and downstream transcriptional programs. The non-synonymous SNP rs16969968 (G>A) in CHRNA5 results in an aspartic acid to asparagine substitution at residue 398 (D398N), a change reported to alter receptor function and intracellular signaling dynamics (Chaity and Apu, 2022). Although this variant has been associated with nicotine dependence and increased susceptibility to smoking-related diseases, its direct immunomodulatory consequences in COPD remain insufficiently defined. Matrix metalloproteinase-9 (MMP-9) is a pivotal immune effector molecule implicated in extracellular matrix degradation, leukocyte recruitment and inflammatory amplification within the pulmonary microenvironment (Baltazar-García et al., 2024). Elevated MMP-9 activity contributes to emphysematous tissue destruction and airway remodeling, hallmark features of COPD (Augoff et al., 2022). However, whether genetic variation in CHRNA5 influences MMP-9 expression and thereby modulates inflammatory remodeling has not been comprehensively examined.

Given the limited data from Indian populations and the need to clarify the immune-genetic mechanisms underlying COPD, the present study investigates the association of the CHRNA5 rs16969968 polymorphism with disease susceptibility and evaluates its relationship with MMP-9 expression levels in the West Bengal population. By integrating genetic association analysis with functional and computational approaches, this study aims to elucidate how variation in a cholinergic receptor gene may shape inflammatory protease activity and contribute to COPD pathogenesis.

## 2 Materials and methods

### 2.1 Study participants and sample collection

This study was designed as a hospital based case-control study. COPD cases and controls were matched primarily based on age and sex to minimize potential confounding effects. The study was approved by the Clinical Research Ethics Committee of the Allergy and Asthma Research Centre, West Bengal, India (CREC-AARC Ref: 65/206), included 412 COPD patients and 390 controls aged between 41–80 years who provided written informed consent. A statistical power of >80% was achieved, indicating that the sample size was sufficient to provide adequate statistical strength for detecting genetic association. COPD severity was classified using post-bronchodilator FEV1/FVC ratio <0.70 according to GOLD 2025 criteria (Global Initiative for Chronic Obstructive Lung Disease GOLD, 2025) and post-bronchodilator FEV1 percentage values according to GOLD 2025 classification. Participants with a history or clinical evidence of asthma, tuberculosis, interstitial lung disease, lung cancer, acute respiratory infection and systemic inflammatory disorders were excluded based on available clinical evaluation and medical records.

### 2.2 SNP selection and genotypic analysis

To conduct this study CHRNA5 rs16969968 SNP was selected using SNPpedia database. Peripheral blood (2 mL) was collected in EDTA tubes, centrifuged, and stored at −20 °C, and genomic DNA was extracted using the QIAamp DNA Blood MiniKit (Qiagen, Germany). The CHRNA5 rs16969968 polymorphism was genotyped using PCR-RFLP method. Primer details and conditions are summarized in Table 1. The RFLP findings of each genotype were validated using Sanger sequencing (Applied Biosystems, USA).

**TABLE 1:** PCR Conditions and restriction fragments of CHRNA5 gene polymorphism.

SNP	Position	Primer sequence and PCR product size	PCR condition	Restriction fragments and genotypes
CHRNA5 (rs16969968)	Exon 5: D398N aspartic acid to asparagine	Forward : 5′-CGCCTTTGGTCCGCAAGATA-3′ Reverse: 5′-TGCTGATGGGGGAAGTGGAG-3^’^ Product size:435bp	95 °C for 5 min, followed by 30 cycles at 95 °C for 30 s, 62 °C for 30 s, 72 °C for 1 min, and a final extension at 72 °C for 10 min	Enzyme: TaqI GG: 290, 145 bp GA: 435,290,145 bp AA: 435 bp

### 2.3 Bronchial epithelial cell (BECs) and bronchoalveolar lavage fluid (BALF) collection via bronchoscopy

BALF and bronchial epithelial cell (BEC) samples were obtained through bronchoscopy in both COPD patients and controls. Total number of sample collected n = 158 (patient 90 and control 68). Sample collection was performed in both patients and controls using the same standardized protocol and comparable anatomical sampling sites to minimize procedural variability. The control samples were taken from patient who had bronchiectasis and were admitted to the hospital with symptoms of hemoptysis but were subsequently confirmed to be free from COPD. In patient group the number of sample for each genotype were GG:30, GA:30, AA:30 whereas in control group the number of sample for each genotype was GG:30 GA:30 AA:8. Furthermore, the patient group were divided into smoker patients (n = 60; GG: 20, GA:20, AA:20) and non-smoker patients (n = 30; GG:10, GA:10, AA:10). Similarly control participants were divided into smoker control (n = 46; GG:20, GA:20, AA:6) and non-smoker control (n = 22; GG:10, GA:10, AA:2). Subgroup analyses were performed to evaluate the functional relevance of the rs16969968 polymorphism beyond its association with COPD susceptibility. The harvested cells were loosened by thorough agitation, passed through a 70-μm mesh to eliminate mucus and debris, and centrifuged at 14,000 rpm for 10 min at 4 °C. The resulting cell pellet was then reconstituted in PBS for downstream RNA and protein isolation. The qPCR, Western blot, and ELISA were performed on the same set of participants. BECs were fixed and subsequently stained with Giemsa (Sigma-Aldrich; 1:20) to evaluate cellular morphology. After staining, the cells were mounted using DPX and visualized under a light microscope at 40× magnification.

### 2.4 Real-time quantitative PCR

Bronchial epithelial cells (BECs) obtained from COPD patients (n = 90) and controls (n = 68) were processed for RNA extraction. Cells were resuspended in 1× PBS, and total RNA was isolated using TRIzol reagent (Invitrogen, USA) following the manufacturer’s protocol. RNA concentration and purity were measured with a NanoDrop spectrophotometer (Thermo Fisher), and integrity was confirmed by agarose gel electrophoresis. To eliminate residual genomic DNA, samples were treated with DNase I (Qiagen) prior to cDNA synthesis. Relative expression of CHRNA5 and MMP9 gene for each genotype of CHRNA5 rs16969968 polymorphism was determined using Hi-Quanti One Step SYBr-Based RT-PCR Kit (HiMedia, India) following manufacturer’s protocol. GAPDH mRNA served as the endogenous control and the primer details were given in Table 2. The stability of GAPDH expression was evaluated across study groups prior to normalization. No significant variation in GAPDH ct values was observed between cases and controls, supporting its use as the endogenous reference gene. Primer validation was performed prior to expression analysis. Amplification efficiencies for CHRNA5, MMP9 and GAPDH were 98%. Melt curve analysis demonstrated single peaks without primer dimer formation. qRT-PCR reactions were run in triplicate wells per biological sample. Technical replicate values were averaged before statistical analysis. Relative gene expression across three genotypes were quantified using mRNA expression fold change 2^−ΔΔCq^ method (Livak and &Schmittgen, 2001; Ganai et al., 2025).

**TABLE 2:** Primer sequences used for quantitative real-time PCR (RT-qPCR) analysis of CHRNA5, MMP-9 and GAPDH.

Gene	Forward primer	Reverse primer
CHRNA5	5′-ACG​TCT​GGT​TGA​AAC​AGG​AAT​G-3′	5′-TGT​AGT​TTG​CCG​GTG​GAG​TC-3′
MMP-9	5′-GAG​TGG​CAG​GGG​GAA​GAT​GC-3′	5′-CCT​CAG​GGC​ACT​GCA​GGA​TG-3′
GAPDH	5′-TTG​ATT​TTG​GAG​GGA​TCT​CGC​TC-3′	5′- GAG​TCA​ACG​GAT​TTG​GTC​GTA​TTG-3′

### 2.5 Western blot analysis

Through bronchoscopy Bronchial epithelial cell (BECs) were collected from patient (n = 90) and control participants (n = 68). Total cellular protein was extracted BECs using RIPA buffer. Protein concentration was quantified using the Bradford assay. Equal concentration of protein of each genotype, loaded in triplicate, were separated by SDS-PAGE based on molecular mass and transferred onto a nitrocellulose membrane. Membranes were blocked with 5% BSA and incubated overnight with primary antibodies: Anti-CHRNA5 rabbit polyclonal antibody (ABclonal, 1:2000) and anti-GAPDH rabbit polyclonal antibody (Bio Bharati Life Science, 1:2000). Detection was carried out using HRP-conjugated secondary antibody (ABclonal, 1:5000). Band intensities were analyzed via densitometry using ImageJ software.

### 2.6 ELISA

BALF of the COPD patient (n = 90) and control (n = 68) participants were collected after bronchoscopy and stored at 4 °C for further use. The level of MMP-9 protein in BALF was quantified using MMP-9 ELISA kit (Krishgen Biosystems, India) according to manufacturer’s protocol.

### 2.7 *In-silico* analysis

#### 2.7.1 Prediction of evolutionary conservancy and secondary structure of CHRNA5

The evolutionary conservation of CHRNA5 was evaluated using the ConSurf web-server (Celniker et al., 2013) which estimated residue conservation based on phylogenetic relationships among homologous sequences employing an empirical Bayesian inference approach. In addition to conservation scores, ConSurf also predicted the residues of CHRNA5 to be exposed or buried. The secondary structure of CHRNA5 was predicted using the PSIPRED tool (Buchan and Jones, 2019) which utilized two feed-forward neural networks to analyse the output generated by PSI-BLAST.

#### 2.7.2 Prediction of functional consequences of SNP on CHRNA5

The functional impacts of SNP (rs16969968) on CHRNA5 was predicted using the following sequence based methods such as FATHMM-XF (Shihab et al., 2012), Mutation Assessor, PANTHER (Tho et al., 2021), IMutant3.0 (Capriotti et al., 2005), MUpro (Cheng et al., 2005) and PhD-SNP (Capriotti et al., 2006). FATHMM-XF, a bioinformatics tool under FATHMM web-server predicted the functional consequences of non-coding and coding single nucleotide variants (SNVs).

#### 2.7.3 Structure modelling and 3D assessment

Template guided homology modelling of CHRNA5 was performed using I-TASSER (Zheng et al., 2021), Phyre2.2 (Powell et al., 2025) and SwissModel (Bienert et al., 2017) web-servers. The modelled structures were validated by Ramachandran plots obtained from PROCHECK (Laskowski et al., 1996).

#### 2.7.4 Impact of SNP on structural deviation and stability of CHRNA5

The structural deviation of CHRNA5 due to SNP induced amino acid substitution was estimated using TM-align web-server (Zhang and Skolnick, 2005). The server gives output in the form TM score for estimation of topological variation and RMSD (Å) for structural deviation analyses of protein due to amino acid substitution. The structural stability of CHRNA5 upon amino acid variation was measured using DynaMut2 (Rodrigues et al., 2021), mCSM (Pires et al., 2014a), INPS-MD (Savojardo et al., 2016), DUET (Pires et al., 2014b) and SAAFEC-SEQ (Li et al., 2021) web-servers. SAAFEC-SEQ employed a gradient boosting decision tree machine learning approach to estimate the change in folding free energy resulting from amino acid substitutions in proteins. DynaMut2 assessed changes in stability and flexibility upon missense mutations.

#### 2.7.5 Effect of SNP on the alteration of microRNAs induced CHRNA5 gene expression

As the SNP of interest is located within the coding sequence (CDS) of gene, we have retrieved the microRNAs (miRNAs) that target the CDS of CHRNA5 gene using miRDb database (Chen and Wang, 2020). The binding sites and binding affinities of the miRNAs were determined for both wild type and polymorphic CDS of CHRNA5 using RNA22 v2 web-server (Hofacker et al., 1994). The optimal secondary structure and tertiary structure of miRNA-target (CHRNA5 mRNA) complex was determined using RNAfold (Gruber et al., 2008) and RNA COMPOSURE (Sarzyńska et al., 2023) web-servers respectively.

### 2.8 Statistical analysis

Categorical data were expressed as numbers (percentages) and continuous variables as mean ± SD. Group comparisons were performed using Pearson’s chi-square test (categorical) and independent t-test (continuous). Genotype and allele frequencies were compared by contingency chi-square and risk associations were assessed using odds ratios (OR) under additive, recessive and dominant models. Hardy-Weinberg equilibrium (HWE) in controls was tested by chi-square. Multivariable logistic regression analyses were performed to evaluate the independent association between rs16969968 genotype and COPD susceptibility under additive, dominant, and recessive genetic models. COPD status was included as the dependent variable, while rs16969968 genotype served as the independent variable. The analyses were adjusted for age, sex, smoking status, pack-years, smoking duration and cigarettes per day as covariates. The Generalized Linear model (GLM) was done by using binomial family and logit link. Data distribution was assessed for normality using the Shapiro-Wilk test. Associations of FEV1 and FEV1/FVC with CHRNA5 genotypes in smoker and non-smoker patients were analyzed by one-way ANOVA followed by *post hoc* Tukey’s test with Bonferroni correction. Linear regression evaluated relationships of smoking duration, pack-years and cigarettes per day with FEV1/FVC. mRNA expression (CHRNA5, MMP-9) and protein levels (CHRNA5, BALF MMP-9) across CHRNA5 rs16969968 genotypes were compared using one-way ANOVA followed by *post hoc* Tukey’s test with Bonferroni correction. To account for potential confounding factors, multivariable linear regression analyses were performed for CHRNA5 and MMP9 expression outcomes. Expression levels were treated as dependent variables, while genotype was included as the independent variable. Age, sex, smoking status, pack-years, COPD severity and COPD status were included as covariates in the adjusted models. All analyses were performed in GraphPad Prism v5 (San Diego, CA), with p < 0.05 considered significant.

## 3 Results

### 3.1 Demographic and clinical characteristics

The information regarding demographic and clinical characteristics of patients and control subjects are depicted in Table 3. No significant difference was observed between patient and control groups in terms of age, sex, family history, residential status and occupation. However, marked disparity was identified in smoking habit with a significantly higher prevalence of smoking among patients as compared to controls (P = 0.001). Significant difference was found between smoker patient and control group in terms of smoking duration (P = 0.01), pack years (P = 0.00008) and cigarettes per day (P = 0.015). Both male and female patients exhibited significantly lower FEV1 and PEFR values relative to predicted values.

**TABLE 3:** Demographic and Clinical parameters of case and control subjects.

Parameters	Category	Patients (n = 412)	Control (n = 390)	P value
Age	41–50	138 (33.49%)	168 (43.07%)	0.10
	51–60	122 (29.61%)	122 (31.28%)	
	61–70	79 (19.17%)	74 (18.97%)	
	71–80	73 (17.71%)	26 (6.66%)	
Sex	Male	214 (51.9%)	200 (51.37%)	0.88
	Female	198 (48.05%)	190 (48.62%)	
Family history	Paternal	68 (16.55%)	Nil	—
	Maternal	80 (19.41%)	Nil	
	P + M	71 (17.23%)	Nil	
	Absent	193 (46.84%)	390 (100%)	
Height (cm)	Male	167.12 ± 6.08 (166.30–167.94)	168.07 ± 5.02 (167.36–168.77)	0.44
	Female	162.14 ± 5.73 (161.33–162.94)	160.33 ± 5.36 (159.56–161.10)	
Weight (kg)	Male	65.38 ± 7.21 (64.41–66.36)	66.64 ± 6.34 (65.75–67.52)	0.62
	Female	62.07 ± 8.47 (60.88–63.26)	61.93 ± 8.34 (53.13–55.91)	
Residence	Urban	160 (38.83%)	152 (38.97%)	0.85
	Semi-urban	155 (37.62%)	136 (34.87%)	
	Rural	97 (23.54%)	102 (26.15%)	
Occupation	Industry	198 (48.05%)	144 (36.92%)	0.22
	Business	126 (30.58%)	121 (31.02%)	
	Agriculture	20 (4.85%)	40 (10.25%)	
	Housewife	50 (12.13%)	46 (11.79%)	
	Unemployed	18 (4.36%)	39 (10.00%)	
GOLD stage (%FEV1)	GOLD 1 (≥80%) (Mild)	104 (25.24%)	Nil	—
	GOLD 2 (50%–79%) (Moderate)	112 (27.18%)	Nil	
	GOLD 3 (30%–49%) (Severe)	122 (29.61%)	Nil	
	GOLD 4 (<30%) (Very severe)	74 (17.965)	Nil	
Smoking habit	Currently smoking	156 (37.86%)	103 (26.41%)	0.001*
	Ex-smoker	92 (22.33%)	62 (15.89%)	
	Non- smoker	164 (39.80%)	225 (57.69%)	
Smoking duration (Years)	1–10	22 (8.87%)	31 (18.78%)	0.01*
	11–20	24 (9.67%)	32 (19.39%)	
	21–30	54 (21.77%)	40 (24.24%)	
	31–40	72 (29.03%)	34 (20.60%)	
	40 and above	76 (31.64%)	28 (16.96%)	
Pack years	1–10	20 (8.06%)	44 (26.66%)	0.00008*
	11–20	38 (15.32%)	46 (27.87%)	
	21–30	64 (25.80%)	32 (19.39%)	
	31–40	71 (28.62%)	28 (16.96%)	
	41and above	55 (22.17%)	15 (9.09%)	
Cigarette per day	1–10	64 (25.8%)	63 (38.18%)	0.015*
	10–20	78 (31.45%)	62 (37.57%)	
	>20	106 (42.74%)	40 (24.24%)	
PEFR(male) (L/min)	Predicted^a^	431.43 ± 26.58 (428.01–435.17)	439.16 ± 24.40 (435.76–442.57)	0.00001*
	Actual	218.11 ± 40.76 (212.90–223.86)	397.01 ± 28.99 (392.97–401.05)	
PEFR(female) (L/min)	Predicted^b^	301.30 ± 20.79 (298.31–304.29)	300.39 ± 19.57 (297.59–303.19)	
	Actual	192.74 ± 40.78 (187.02–198.45)	285.62 ± 21.68 (282.52–288.73)	
FEV1(male)	Predicted^c^	2.61 ± 0.30 (2.56–2.65)	2.69 ± 0.27 (2.65–2.73)	0.00001*
	Actual	1.46 ± 0.17 (1.43–1.48)	2.51 ± 0.31 (2.47–2.55)	
FEV1(female)	Predicted^d^	2.09 ± 0.24 (2.06–2.12)	2.1 ± 0.22 (2.07–4.18)	
	Actual	1.39 ± 0.17 (1.36–1.41)	1.87 ± 0.22 (1.84–1.90)	
FEV1/FVC	—	0.44 ± 0.07 (0.43–0.45)	0.79 ± 0.06 (0.78–0.80)	0.00001*

* P value significant.

^a^ Predicted PEFR, for male (L/min) = −1.807*age +3.206*height in cm.

^b^ Predicted PEFR, for female (L/min) = −1.454*age +2.368*height in cm.

^c^ Predicted FEV1 for male (L) = −1.7649+ (−0.0218*age) + (0.0337*height in cm).

^d^ Predicted FEV1 for female (L) = 0.0381+ (−0.0197*age) + (0.0196*height in cm) (Ganai et al., 2023).

### 3.2 Genotypic analysis of CHRNA5 rs16969968 in the case-control study

**FIGURE 1:**
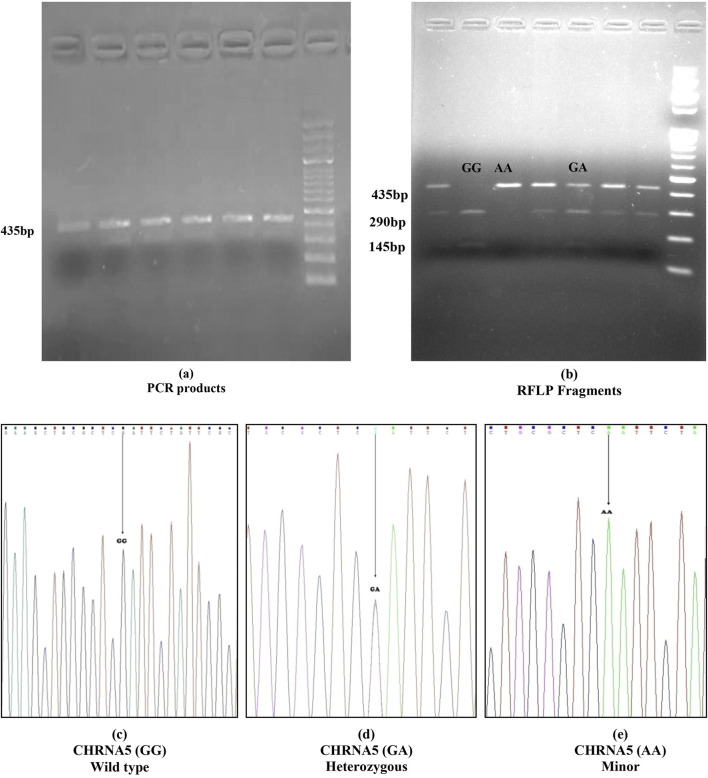
Genotypic result of CHRNA5 rs16969968 polymorphism: **(a)** Represents PCR products respectively. The last lane shows the 100bp ladder. **(b)** Represents RFLP fragments respectively. **(c–e)** Denote representative chromatograms respectively, position of the genotypes is shown with an arrow.

Information regarding PCR-RFLP of each genotype is given in Figure 1. The overall genotyping call rate was 100% (802/802). A subset of samples (11.22%, n = 90) was validated by Sanger sequencing, showing 100% concordance with PCR-RFLP results. The digestion efficiency was 100% with <1% error rate. Population stratification was carefully addressed by selecting representative patient and control samples from all predefined stratified subgroups. Genotyping was performed blinded to case/control status. Duplicate samples were not included. Genotypic and allelic distribution of CHRNA5 rs16969968 is summarized in Table 4. Controls showed no deviation from Hardy–Weinberg equilibrium (χ^2^ = 1.067 at df = 1). Genotype frequencies differed significantly between patients and controls under additive (P = 0.001), recessive (P = 0.009), and dominant (P = 0.002) models. The AA genotype was more frequent in patients under additive (OR: 8.07, 95% CI: 1.71–38.10, P = 0.008), recessive (OR: 6.05, 95%CI: 1.30–28.07, P = 0.02) and dominant models (OR: 2.41, 95% CI: 1.35–4.29, P = 0.002). Allele A was significantly enriched in patients (OR: 2.29, 95%CI: 1.17–4.48, P = 0.014). Genotypic distribution of CHRNA5 rs16969968 by smoking status presented in Table 5, genotype distribution varied significantly with smoking status (P = 0.0001). Smoker patients carrying the AA genotype had higher COPD risk than smoker (OR = 5.52, 95% CI = 1.71–17.83 P = 0.004) and non-smoker controls (OR = 20.84, 95% CI = 2.64–164.14, P = 0.003), while GA smokers also showed increased risk (OR = 2.58, 95% CI = 1.38–4.81, P = 0.002; OR = 1.9, 95% CI = 1.05–3.46, P = 0.034). Association of genotype frequency with smoking duration (P < 0.0001), pack-years (P < 0.0001), and cigarettes per day (P < 0.0001) mentioned in Table 6. AA carriers with 11–20, 21–30, 31–40, and >40 years of smoking (OR = 4.96, 95% CI = 1.32–18.54, P = 0.017; OR = 10.34, 95% CI = 2.22–48.03, P = 0.01; OR = 6.9, 95% CI = 1.84–25.82, P = 0.004; OR = 77, 95% CI = 4.52–1310.6, P = 0.002 respectively) and with 11–20, 21–30, 31–40, and >40 pack-years (OR = 4.83, 95% CI = 1.48–15.78, P = 0.009; OR = 6.63, 95% CI = 1.78–24.74, P = 0.004; OR = 8.91, 95% CI = 2.43–32.66, P = 0.001; OR = 76.17, 95% CI = 4.48–1294.5, P = 0.002 respectively) showed significant risk. Similarly, AA genotype was associated with smoking 10–20 and >20 cigarettes/day (OR = 12.04, 95% CI = 2.61–55.55, P = 0.0014; OR = 15.11, 95% CI = 3.33–68.40, P = 0.0004 respectively).

**TABLE 4:** Genotypic frequency distribution of CHRNA5 rs16969968 polymorphism among case control subjects.

Genotype/Allele	Model	Patients (N = 412)	Control (N = 390)	Chi square value (P value)	Odds ratio (95% CI)	P value	MAF
GG	Additive	196 (47.57%)	268 (68.71%)	12.78 (0.001*)	1	-	0.2
GA		172 (41.74%)	114 (29.23%)		2.12 (1.16–3.87)	0.014*	
AA		44 (10.67%)	8 (2.05%)		8.07 (1.71–38.10)	0.008*	
GG + GA	Recessive	368 (89.32%)	382 (97.95%)	6.66 (0.009*)	1	-	
AA		44 (10.67%)	8 (2.05%)		6.05 (1.30–28.07)	0.02*	
GG	Dominant	196 (47.57%)	268 (68.71%)	9.08 (0.002*)	1	-	
GA + AA		216 (52.42%)	122 (31.28%)		2.41 (1.35–4.29)	0.002*	
G	-	564 (68.44%)	650 (83.33%)	6.08 (0.013*)	1	-	
A	-	260 (31.55%)	130 (16.66%)		2.29 (1.17–4.48)	0.014*	

**TABLE 5:** Genotypic frequency distribution of CHRNA5 rs16969968 according to smoking habit.

Genotype	Smoker patient (n = 248)	Non-smoker patient (n = 164)	Smoker control (n = 165)	Non-smoker control (n = 225)	Chi square value (p value)	OR (95% CI)				P value				MAF
						A	B	C	D	A	B	C	D	
GG	112 (45.16%)	84 (52.21%)	118 (71.51%)	150 (66.66%)	28.08 (0.0001*)	1	1	1	1	-	-	-	-	0.2
GA	102 (41.12%)	70 (42.68%)	41 (24.84%)	73 (32.44 %)		2.58 (1.38-4.81)	1.9 (1.05-3.46)	0.79 (0.43-1.45)	0.73 (0.39-1.37)	0.002*	0.034*	0.45	0.33	
AA	34 (13.70%)	10 (6.09%)	6 (3.63%)	2 (0.88%)		5.52 (1.71-17.83)	20.84 (2.64-164.14)	2.04 (0.55-7.62)	3.77 (0.41-34.63)	0.004*	0.003*	0.2	0.24	

**TABLE 6:** Genotypic frequency distribution of CHRNA5 rs16969968 according to smoking related parameters.

Genotype	Patient Smokers (n = 248)					Control Smokers (n = 165)					Chi square Value (P value)	Odds ratio (95% CI)					P value					MAF
I. Distribution according to smoking duration (years)																						
	1–10 years (n = 22)	11–20 years (n = 24)	21–30 years (n = 54)	31–40 years (n = 72)	41 years-above (n = 76)	1–10 years (n = 31)	31–40 years (n = 32)	21–30 years (n = 40)	11–20 years (n = 34)	41 years-above (n = 28)	112.5 (0.0001*)	1–10 years	11–20 years	21–30 years	31–40 years	41 years -above	1–10 years	11–20 years	21–30 years	31–40 years	41 years -above	0.2
GG	12 54.54%)	12 (50.00%)	24 (44.44%)	32 (44.44%)	32 (42.10%)	18 (58.06%)	20 (62.5%)	28 (70.00%)	26 (76.47%)	26 (92.85%)		1	1	1	1	1	-	-	-	-	-	
GA	8 (36.36%)	9 (37.5%)	23 (42.59%)	31 (43.05%)	31 (40.78%)	10 (32.25%)	11 (34.37%)	11 (27.50%)	7 (20.58%)	2 (7.14%)		1.20 (0.66–2.20)	1.34 (0.74–2.44)	2.47 (1.34–4.5)	3.53 (1.94–7.09)	12.96 (5.37–31.28)	0.53	0.32	0.003*	0.0001*	0.0001*	
AA	2 (9.09)	3 (12.5%)	7 (12.96%)	9 (12.5.%)	13 (17.10%)	3 (9.67%)	1 (3.12%)	1 (2.5%)	1 (2.94%)	0 (0%)		0.96 (0.36–2.55)	4.96 (1.32–18.54)	10.34 (2.22–48.03)	6.9 (1.84–25.82)	77.0 (4.52–1310.64)	0.94	0.017*	0.01*	0.004*	0.002*	
II. Distribution according to pack years																						
	1–10 (n = 20)	11–20 (n = 38)	21–30 (n = 64)	31–40 (n = 71)	41- above (n = 55)	1–10 (n = 44)	1–20 (n = 46)	21–30 (n = 32)	31–40 (n = 28)	41- above (n = 15)	137.7 (0.0001*)	1–10	11–20	21–30	31–40	41- above	1–10	11–20	21–30	31–40	41- above	0.2
GG	10 (50.00%)	17 (44.73%)	30 (46.87%)	32 (45.07%)	23 (41.81%)	25 (56.81%)	31 (67.39%)	25 (78.12%)	24 (85.71%)	13 (86.66%)		1	1	1	1	1	-	-	-	-	-	
GA	9 (45.00%)	16 (42.10%)	26 (40.62%)	29 (40.84%)	22 (40.00%)	17 (38.63%)	13 (28.26%)	6 (18.75%)	3 (10.71%)	2 (13.33%)		1.31 (0.74–2.33)	2.23 (1.21–4.10)	3.58 (1.86–6.88)	7.12 (3.34–15.18)	6.37 (3.08–13.17)	0.34	0.009*	0.0001*	0.0001	0.0001*	
AA	1 (5.00%)	5 (13.15%)	8 (12.5%)	10 (14.08%)	10 (18.18%)	2 (4.54%)	2 (4.34%)	1 (3.125)	1 (3.57%)	0 (0%)		1.42 (0.36–5.59)	4.83 (1.48–15.78)	6.63 (1.78–24.74)	8.91 (2.43–32.66)	76.17 (4.48–1294.55)	0.61	0.009*	0.004*	0.001*	0.002*	
III. Distribution according to cigarettes per day																						
	1–10 (n = 64)		10–20 (n = 78)		>20 (n = 106)	1–10 (n = 63)			10–20 (n = 62)		>20 (n = 40)	59.74 (0.0001*)		1–10 (n = 64)	10–20 (n = 78)	>20 (n = 106)	1–10 (n = 63)		10–20 (n = 62)		>20 (n = 40)	0.2
GG	30 (46.8%)		34 (43.58%)		48 (45.28%)	35 (55.55%)			47 (74.19%)		37 (80%)			1	1	1	-		-		-	
GA	29 (45.31%)		33 (42.30%)		40 (37.73%)	24 (38.09%)			14 (24.19%)		2 (17.5%)			1.58 (0.89–2.81)	3.01 (1.60–5.63)	3.97 (2.01–7.83)	0.27		0.0006*		0.0001*	
AA	5 (7.8%)		11 (14.10%)		18 (16.98%)	4 (6.34%)			1 (1.61%)		1 (2.5%)			1.56 (0.5.-4.81)	12.04 (2.61–55.55)	15.11 (3.33–68.40)	0.43		0.0014*		0.0004*	

• Significant association shown in asterisk*.

• Abbreviation: CI, confidence interval; MAF, Minor allele frequency in control group; OR- Odds ratio.

A-OR, against Smoker patients vs. Smoker controls and its corresponding P value.

B-OR, against Smoker patients vs. Non-smoker controls and its corresponding P value.

C-OR, against Non-smoker patients vs. Smoker controls and its corresponding P value.

D-OR, against Smoker controls vs. non-smoker controls and its corresponding P value.

The multivariable logistic regression analyses demonstrated that the rs16969968 polymorphism remained significantly associated with COPD susceptibility under additive (adjusted OR:2.40, 95%CI: 1.48–3.94, P value: 0.0004) dominant (adjusted OR:2.48; 95% CI: 1.40–4.48, P value: 0.0027) and recessive (adjusted OR: 6.11, 95% CI: 1.97–22.64, P value: 0.0044) genetic models even after adjustment for age, sex, smoking status, pack-years, smoking duration and cigarettes per day (Supplementary Table 1). GLM result showed rs16969968 genotype is significantly associated with smoking duration (p < 0.0004), pack years (p < 0.002) and cigarettes per day (0.0006) after adjusting.

## 4 Lung function and COPD

**FIGURE 2:**
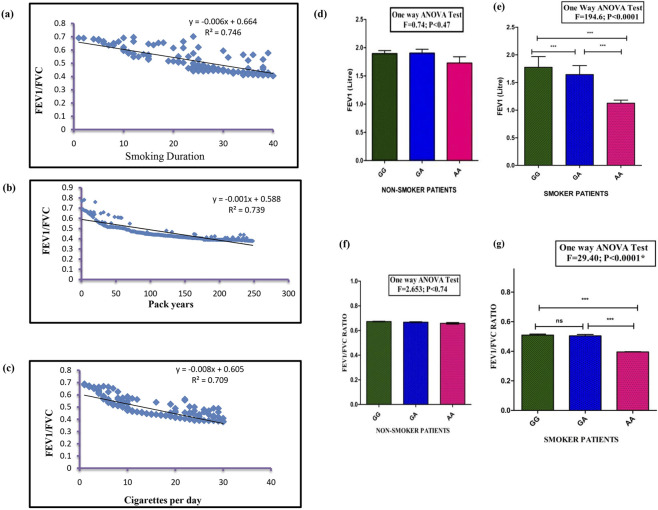
Association between different smoking parameters and lung function (FEV1, FEV1/FVC ratio): **(a)** FEV1/FVC ratio with smoking duration (years), **(b)** Pack years with FEV1/FVC ratio. **(c)** Cigarette per day with FEV1/FVC ratio in smoker COPD patients. **(d)** Level of FEV1 in non-smoker patients. Comparison between different genotypes of rs16969968 was performed by One Way ANOVA Test followed by Post hoc Tukey analysis with Bonferroni correction. **(e)** Level of FEV1 in smoker patients bearing different genotypes of CHRNA5 rs16969968. Comparison between different genotypes was performed by One Way ANOVA Test followed by Post hoc Tukey analysis with Bonferroni correction. **(f)** FEV1/FVC ratio in non-smokers. Comparison between different genotypes of rs16969968 was performed by One Way ANOVA Test followed by Post hoc Tukey analysis with Bonferroni correction. **(g)** FEV1/FVC ratio in smoker patients bearing different genotypes for CHRNA5 rs16969968. Comparison between different genotypes was performed by One Way ANOVA Test followed by Post hoc Tukey analysis with Bonferroni correction. Statistical significance is indicated as p > 0.05 (ns) p < 0.05 (*), p < 0.01 (**), and p < 0.001 (***).

The relationship between FEV1/FVC ratio and smoking-related parameters is shown in Figures 2a–c. Significant inverse relationship was observed for FEV1/FVC ratio with smoking duration (R^2^ = 0.746), pack-years (R^2^ = 0.739) and cigarettes per day (R^2^ = 0.709). Association of CHRNA5 rs16969968 with FEV1 presented in Figures 2d,e. One-way ANOVA showed significantly reduced values in smoker patients with AA genotype compared to GG and GA (F = 194.6, P < 0.0001) after Bonferroni correction. Association of CHRNA5 rs16969968 with FEV1/FVC ratio illustrated in Figures 2f,g. One-way ANOVA showed significantly reduced values in smoker patients with AA genotype compared to GG and GA (F = 29.40, P < 0.0001) after Bonferroni correction. However, no significant differences were observed in non-smoker patients (P > 0.05).

### 4.1 Prediction of CHRNA5 and MMP9 expression in BECs

**FIGURE 3:**
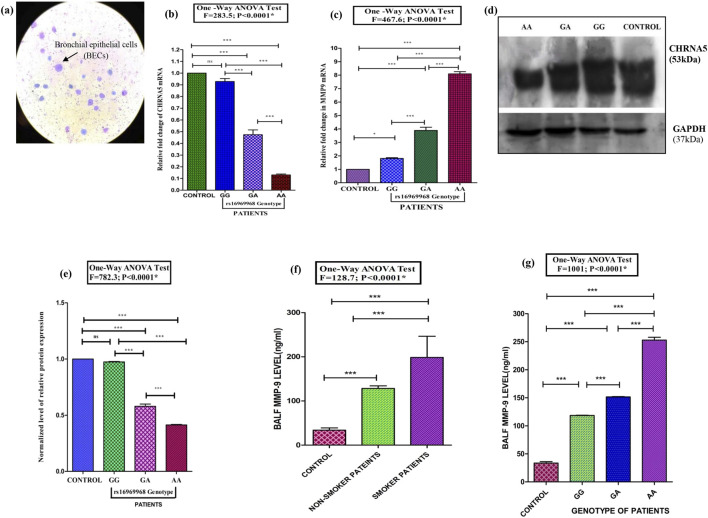
CHRNA5 and MMP-9 expression in bronchial epithelial cell and BALF: **(a)** Giemsa staining reveals characteristic epithelial morphology, and well defined nuclei of BECs (40x). **(b)** Represents mRNA expression level of CHRNA5 using 2^ΔΔCq^ method in control and patients bearing different genotypes of CHRNA5 rs16969968. Comparison between different genotypes in case and control was performed by One Way ANOVA Test followed by Post hoc Tukey analysis with Bonferroni correction. **(c)** Represents mRNA expression level of MMP-9 using 2^-ΔΔCq^ method in control and patients bearing different genotypes of CHRNA5 rs16969968. Comparison between different genotypes in case and control was performed by One Way ANOVA Test followed by Post hoc Tukey analysis with Bonferroni correction. **(d)** Representative image of Western blot analysis of CHRNA5 in different polymorphic genotypes of case and control. Human GAPDH protein was used as loading control. The blot shown is representative of independent biological replicates. **(e)** Protein expression of CHRNA5 in control and different polymorphic genotype of patients. Comparison between different genotypes in case and control was performed by One Way ANOVA Test followed by Post hoc Tukey analysis with Bonferroni correction. **(f)** Level of MMP-9 in BALF of control, smoker and non-smoker COPD patients. Comparison between different controls, smoker and non-smoker COPD patients was performed by One Way ANOVA Test followed by Post hoc Tukey analysis with Bonferroni correction. **(g)** MMP-9 level in BALF of COPD patients bearing different genotype of CHRNA5 rs16969968. Comparison between different genotypes in case and control was performed by One Way ANOVA Test followed by Post hoc Tukey analysis with Bonferroni correction. Statistical significance is indicated as p > 0.05 (ns) p < 0.05 (*), p < 0.01 (**), and p < 0.001 (***).

Giemsa staining images of BECs provided an initial morphological assessment of the bronchial epithelial cell monolayer given in Figure 3a. The mRNA expression of CHRNA5 across different genotypes of rs16969968 is shown in Figure 3b. One-way ANOVA indicated significant variation (P = 0.0001). Tukey’s test revealed lower expression in AA and GA patients compared to controls (P < 0.00001), with significant differences among all genotypes except controls vs. GG (P = 0.16) after Bonferroni correction. AA genotype remained significantly associated with reduced CHRNA5 mRNA expression (R^2^ = 0.76, β = −0.21, p < 0.001) after adjusting the potential confounders. Figure 3c depicts MMP9 mRNA expression, which also differed significantly (P < 0.0001). Tukey’s test confirmed significant differences across all genotype comparisons. The expression of MMP9 mRNA was significantly higher in patients having AA risk genotype (P < 0.00001) after Bonferroni correction. Genotype remained significantly associated with increased MMP9 expression (R^2=^ 0.89, β = 0.269, p < 0.001) after adjusting the potential confounders.

### 4.2 Analysis of CHRNA5 protein in COPD

Protein expression of CHRNA5 rs16969968 across different genotypes is shown in Figure 3d. AA risk carriers had significantly lower expression than controls and GG. One-way ANOVA confirmed overall differences (P < 0.0001), and Tukey’s test showed significant variation among all groups (P < 0.00001) except between controls and GG (P = 0.30) after Bonferroni correction, Figure 3e rs16969968 A A genotype remained significantly associated with increased CHRNA5 expression (R^2^ = 0.77, β = −0.07, p < 0.001) after adjusting the potential confounders.

### 4.3 Estimation of MMP-9 level in BALF

Significantly higher MMP-9 levels were found in BALF among smoker and non-smoker patients compared to controls (P < 0.0001), with Tukey’s test confirming group-wise differences (P < 0.00001) presented in Figure 3f. Figure 3g depicts BALF MMP-9 levels across rs16969968 genotypes, with ANOVA showing strong variation (P < 0.0001). Tukey’s test revealed significant differences across all genotype comparisons (P < 0.00001) after Bonferroni correction. rs16969968 A A genotype remained significantly associated with increased MMP-9 expression (R^2^ = 0.82, β = 36.34, p < 0.001) after adjusting the potential confounders.

### 4.4 *In-silico* analysis

To corroborate the findings of wet-lab experimentation regarding rs16969968 (G>A) mediated functional alteration of CHRNA5, we have also performed bioinformatics analyses.

#### 4.4.1 Analyses of evolutionary conservation of SNP site and its effects on secondary structure

**FIGURE 4:**
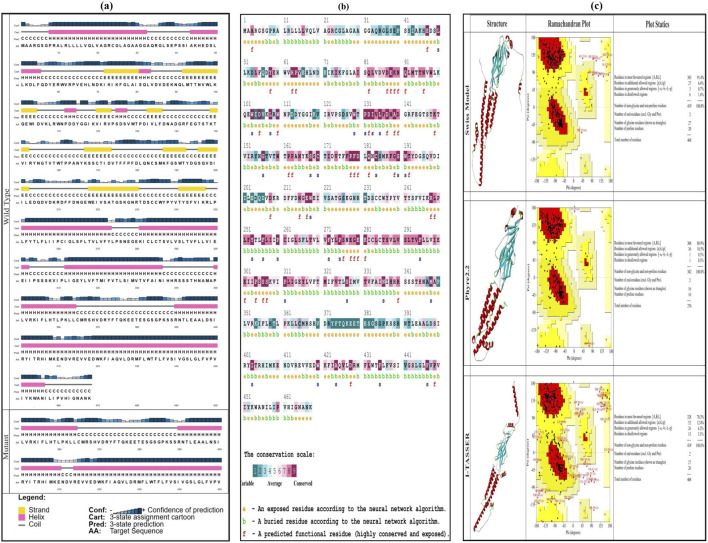
In-silico analysis:**(a)** Evolutionary conservation analyses of CHRNA5 by ConSurf server. Conservation grades of individual amino acid residues are represented by a color-coded scale, indicating varying degrees of evolutionary conservation. **(b)** Prediction of secondary structural details of both wild type and mutant CHRNA5 using the PsiPred server. **(c)** Illustration of structural models, corresponding Ramachandran plots and plot statics predicted by Swiss Model, Phyre2.2 and I-TASSER, respectively.

The evolutionary conservation profile and secondary structure of CHRNA5 were analyzed using the ConSurf (Figure 4a) and PSIPRED servers (Figure 4b), respectively. According to ConSurf analysis, the residue D398 is not highly conserved. We had performed MSA (using Clustal Omega) of CHRNA5 from amino acid sequences of 10 mammals and found that D398 position was highly conserved among them, except for Rabbit (Supplementary Figure 1). This suggests that the D398 position is positively selected during evolution among mammals. Secondary structure analyses of CHRNA5 revealed that D398 is an exposed residue situated within a helical region of the protein. The D398N substitution was found to induce a conformational shift from helix to coil within residues 411–413 (Figure 4b), suggesting a possible local structural alteration. We also found that the polymorphism did not induce any significant alteration in the minimum free energy based mRNA secondary structure of the CHRNA5.

#### 4.4.2 Functional assessment of the impact of D398N variant in CHRNA5

Mutation Assessor predicted a medium functional impact of the SNP on CHRNA5 shown in Table 7. According to PANTHER, the variant is classified as probably damaging. FATHMM-XF, part of the FATHMM web server, predicted the variant to be pathogenic. Both I-Mutant 3.0 and MUpro predicted a decrease in protein stability due to the mutation. Additionally, PhD-SNP indicated a potential disease association for this variant. Collectively, these results suggest that SNP rs16969968 is deleterious, as all six tools consistently predicted an adverse effect on the structure and function of the CHRNA5 protein.

**TABLE 7:** Predicted impact of SNP rs16969968 on CHRNA5 using six sequence-based computational tools.

Web server	Score	Prediction
Mutation assessor	0.659	Medium impact
PANTHER	0.57	Probably damaging
FATHMM-XF	0.686844 (coding score)	Pathogenic
I-mutant 3.0	8 (RI)	Decrease stability
MUpro	−0.39488666 (ΔG)	Decrease stability
PhD-SNP	3	Disease

#### 4.4.3 Homology modeling of CHRNA5 and structural stability analyses of the D398N variant

Due to absence of an experimentally resolved crystal structure of CHRNA5 in protein data bank (PDB), we performed template guided homology modelling of CHRNA5, using modelling platforms. Based on the Ramachandran plot analysis (Figure 4c), the model predicted by Swiss-Model was identified as the most reliable structure and was subsequently subjected to stability analyses. All five web-servers predicted destabilizing nature of D398N amino acid variation shown in Table 8.

**TABLE 8:** Prediction of structural stability changes in CHRNA5 resulting from the D398 N amino acid substitution using five structure based stability prediction tools. Stability was assessed as the change in free energy (ΔΔG), calculated as ΔΔG = ΔG (Variant of Protein) − ΔG (Wild Type Protein), and reported in Kcal/mol.

Server	ΔΔG (Kcal/mol)	Prediction
DynaMut2	−1.13	Destabilising
mCSM	−0.407	Destabilising
INPS-MD	−0.17	Weakly destabilizing
DUET	−0.164	Destabilizing
SAAFEC-SEQ	−0.06	Destabilizing

#### 4.4.4 Analysis of structural and topological deviation

The structural and topological deviations in CHRNA5 caused by the D398N amino acid substitution were assessed using the TM-align web server, by calculating the root-mean-square deviation (RMSD) and TM-score respectively. The analysis revealed that the D398N variant of CHRNA5 exhibits negligible structural and topological alterations, as indicated by an RMSD of 0.04 Å and a TM-score of 0.99998.

#### 4.4.5 Impact of SNP mediated alteration on microRNAs binding to the coding sequence of CHRNA5 mRNA

After assessing the impact of the SNP on the sequence and structural alterations of CHRNA5, we further aimed to investigate whether the variant could be associated with potential miRNA-mediated post-transcriptional regulatory mechanisms. Previous studies have demonstrated that in addition to non-coding regions, miRNAs can also regulate gene expression by targeting the coding sequences of genes (Mondal et al., 2026). We have identified a total of 50 micro RNAs (miRNAs) that can target the coding sequence (CDS) of CHRNA5 mRNA using miRDb database (Table 9). Among the 50 predicted miRNAs, only a few retained binding affinities for both wild type and polymorphic CDS (Table 9). Notably, one miRNA, hsa-miR-2115-5p was predicted to bind specifically to the polymorphic CDS near the SNP locus with a folding energy of −13.5 kcal/mol and p-value of 0.0709. This interaction was absent in the wild type sequence, suggesting that hsa-miR-2115-5p may influence CHRNA5 expression in the presence of rs16969968 variant. As this miRNA-target (polymorphic) gene interactionis not statistically significant, the predicted association between rs16969968 and hsa-miR-2115-5p is presented as a hypothesis-generating observation that may provide a basis for future mechanistic investigations.

**TABLE 9:** Prediction of miRNAs targeting the wild type and polymorphic CDS of *CHRNA5* gene using the RNA22 V2 web-server.

Wild type CDS				Polymorhic CDS		
miRNA name	Leftmost position in CDS binding	Folding energy (Kcal/mol	p value	Leftmost position in CDS binding	Folding energy (Kcal/mol	p value
hsa_miR_1289	12	−13	3.48E-01	12	−13	3.48E-01
hsa_miR_3934_5p	186	−15.2	8.13E-02	186	−15.2	8.13E-02
hsa_miR_4729	236	−13.6	3.77E-01	236	−13.6	3.77E-01
hsa_miR_6740_5p	374	−15.1	3.53E-01	374	−15.1	3.53E-01
hsa_miR_654_3p	378	−12.5	3.53E-01	378	−12.5	3.53E-01
hsa_miR_4676_3p	431	−15	1.50E-01	431	−15	1.50E-01
hsa_miR_7851_3p	441	−16	1.50E-01	441	−16	1.50E-01
hsa_miR_493_3p	455	−12.3	1.50E-01	455	−12.3	1.50E-01
hsa_miR_1289	462	−22	1.50E-01	462	−22	1.50E-01
hsa_miR_664 b_3p	665	−12.2	2.38E-02	665	−12.2	2.38E-02
hsa_miR_634	690	−13.4	2.38E-02	690	−13.4	2.38E-02
hsa_miR_7_5p	795	−13.8	1.22E-01	795	−13.8	1.22E-01
hsa_miR_6740_5p	812	−14.2	1.22E-01	812	−14.2	1.22E-01
hsa_miR_3934_5p	846	−14.2	2.22E-01	846	−14.2	2.22E-01
hsa_miR_1272	896	−12.52	4.89E-02	896	−12.52	4.89E-02
hsa_miR_6740_5p	1066	−19.1	1.89E-01	1066	−19.1	1.89E-01
hsa_miR_503_3p	1144	−19.3	7.09E-02	1144	−19.3	7.09E-02
hsa_miR_503_3p	1250	−12.5	2.66E-01	1250	−12.5	2.66E-01
hsa_miR_7851_3p	1256	−15	2.66E-01	1256	−15	2.66E-01
hsa_miR_2115_5p	-	-	-	1162	−13.5	7.09E-02

## 4 Discussion

Chronic obstructive pulmonary disease (COPD) is a prevalent respiratory disorder marked by irreversible, progressive airflow limitation with chronic cough, sputum production, and respiratory discomfort, severely impairing health and quality of life (Xu et al., 2024). The present findings support a contributory role for the CHRNA5 rs16969968 variant in modulating disease risk, particularly under sustained nicotine exposure. The present study revealed that 53.16% patients have a familial history of COPD (Either maternal, paternal or both) highlighting the potential contribution of hereditary genetic factors. This finding supports the role of genetic predisposition in COPD pathogenesis, consistent with previous evidence linking familial aggregation to increased risk. No significant differences in demographic parameters such as age, sex, height, weight, residence, or occupation were observed, thereby reducing the likelihood of confounding. Smoking emerged as the predominant environmental determinant, with parameters including duration, pack-years and cigarettes per day showing significant associations with COPD, in agreement with earlier findings by (Hopkins et al., 2021). The rs16969968 AA polymorphism remained significantly associated with COPD susceptibility after adjustment for age, sex, smoking status, pack-years, smoking duration and cigarettes per day. Smoking-related variables, including pack-years, smoking duration and cigarettes per day, were also independently associated with COPD susceptibility, suggesting the role of smoking in COPD pathogenesis. Together, these results emphasize the interplay between genetic vulnerability and environmental exposure in the development of COPD. Significant reductions in FEV1, FEV1/FVC ratio, and PEFR observed in COPD patients reaffirm the characteristic airflow limitation of the disease, consistent with previous reports in another population (Johns et al., 2014). The rs16969968 AA genotype was found at a higher frequency among COPD patients than controls, confirming its strong association with disease susceptibility. Stratified analysis further revealed that individuals carrying the AA variant, particularly long-term (>20 years) and heavy smokers (>11 pack year; >10 cigarettes per day), exhibited markedly increased vulnerability to COPD. This finding aligns with earlier evidence linking the CHRNA5 rs16969968 variant to nicotine dependence and increased COPD susceptibility (Hopkins et al., 2021). The D398N substitution within the α5 nicotinic acetylcholine receptor (nAChR) subunit is known to alter receptor function and intracellular calcium permeability, thereby influencing downstream immune signaling cascades. Given that nAChRs are expressed in airway epithelial and immune cells, impaired α5-containing receptor function may disrupt cholinergic anti-inflammatory pathways, enhancing susceptibility to chronic inflammation (Scholze and Huck, 2020).

Beyond its established role in smoking behaviour, the rs16969968 variant in CHRNA5 may exert molecular effects relevant to COPD pathogenesis. The AA genotype is significantly associated with reduced CHRNA5 expression and increased MMP9 mRNA expression after adjustment for relevant confounders. This inverse relationship may indicate a possible interaction between altered nicotinic receptor signalling and increased proteolytic activity within the pulmonary microenvironment. Previous study by (Chaudhuri et al., 2013) similarly reported elevated MMP-9 expression in COPD, supporting the current findings. The reduced CHRNA5 protein expression associated with the AA genotype may disrupt α5 nAChR-mediated calcium signalling, which can amplify pro-inflammatory pathways that induce MMP9 transcription. The resultant increase in MMP-9 activity may contribute to extracellular matrix degradation and alveolar damage characteristic of COPD. Furthermore, consistent with reports that nAChRs are expressed in lung epithelial cells (Lam et al., 2016), the rs16969968 variant may impair epithelial repair mechanisms and potentiate oxidative stress responses, independent of nicotine exposure. The present study also demonstrated a significant elevation of BALF MMP-9 levels in COPD patients compared with airway-expression non-COPD bronchiectasis/hemoptysis controls consistent with observations by (Dimic-Janjic et al. 2023). Notably, smokers exhibited higher MMP-9 concentrations than non-smokers, supporting nicotine’s role as a potent inducer of MMP-9 expression. Patients carrying the rs16969968 AA risk genotype displayed further increased MMP-9 expression, suggesting that genetic variation in CHRNA5 may potentiate inflammatory and proteolytic pathways underlying epithelial remodelling in COPD influencing extracellular matrix degradation and emphysematous damage (Bormann et al., 2022). The adjusted analyses demonstrated that genotype remained significantly associated with both CHRNA5 and MMP-9 expression after controlling for these clinical covariates. The AA genotype showed significantly lower CHRNA5 expression compared with the GG genotype, whereas MMP-9 expression remained significantly elevated in AA carriers. Overall, these findings indicate that CHRNA5 dysfunction may be involved in COPD pathogenesis through altered receptor signalling and related inflammatory and proteolytic responses.

In-silico analyses further revealed that rs16969968 represented a deleterious and moderately structural destabilizing variant of the CHRNA5 gene. Although the D398N variant may not induce any major global structural alterations in CHRNA5; but it could influence receptor activity through localized conformational changes, altered molecular interactions, or modulation of downstream signalling pathways. Furthermore, analyses of miRNA mediated post-transcriptional regulation suggested a plausible variant-associated downregulation of CHRNA5 expression through hsa-miR-2115-5p.

**FIGURE 5:**
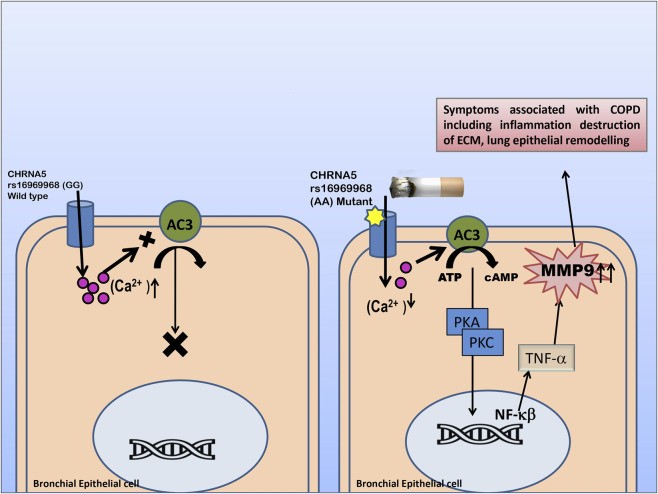
A hypothetical predictive model of CHRNA5-MMP-9 crosslink in COPD regulation: rs16969968 variant reduces nAChR-mediated Ca^2+^ influx and alters bronchial epithelial cell responses and TNF-α expression via an adenylyl cyclase dependent mechanism, ultimately leading to elevated MMP-9 production in COPD.

Based on above findings, a hypothetical predictive model (Figure 5) of COPD regulation via CHRNA5 gene was developed. In rs16969968 wild-type (GG) genotype, elevated intracellular Ca^2+^ inhibits adenylyl cyclase 3 (AC3) activity (Willoughby and Cooper, 2007). Conversely, the rs16969968 variant may decrease Ca^2+^ permeability through nAChRs, which may lead to AC3 over activation and increased cAMP production. This, in turn, may activate PKA, PKC, and NF-κB signalling pathways, which can result as enhanced TNF-α production and subsequent induction of MMP-9, driving extracellular matrix degradation and airway remodelling (Hozumi et al., 2001). Overall, the findings suggest that altered CHRNA5 signalling, together with smoking exposure, may contribute to MMP-9 related lung damage in COPD. From a translational perspective, rs16969968 may represent a potential indicator of increased inflammatory sensitivity to nicotine exposure in COPD. Moreover, Targeting nAChR-mediated signaling pathways or downstream protease activation may provide novel avenues for immunomodulatory intervention in COPD.

Despite the strength of these findings, there are also some limitations of the present study. Firstly, the small study population, which requires more studies in different ethnic groups in different parts of the country to validate the findings. Additionally district-wise genotype frequency analysis was not performed in the present study. Secondly, our study could not include clinical data because of incomplete information availability. Thirdly, due to limited sample availability, some subgroups in the functional analyses, especially the AA genotype control group had small sample sizes. Fourth, the information about biomass-burning smoke “BBS” exposure of COPD patients was not included. Fifth, the role of rs16969968 on CHRNA5 and MMP9 gene expression require further investigation through additional cellular and animal-based studies.

## 5 Conclusion

These findings suggest a possible role of CHRNA5 receptor dysfunction, nicotine exposure and MMP-9 mediated inflammatory lung injury which may contribute to COPD pathogenesis. These findings suggest that the α5 subunit variant may influence host immune responses to nicotine exposure by modulating downstream pro-inflammatory and proteolytic pathways within the pulmonary microenvironment. Its dual association with smoking behavior and COPD-related inflammatory phenotypes suggests that rs16969968 may be a candidate biomarker and hypothesis-generating therapeutic-relevance marker, although further functional validation is required before inferring direct effects on lung pathophysiology or therapeutic targetability. At present the finding is best interpreted as supportive but not definitive, as rs16969968 is a candidate susceptibility marker that merits further replication, functional confirmation and integration into multi-marker risk models rather than immediate clinical use for screening or prognosis.

## Data Availability

The rs16969968 variant information has been deposited in ClinVar under accession SCV007107103. Due to participant privacy and institutional ethical restrictions, the complete de-identified dataset is not publicly available but can be provided by the corresponding author upon reasonable request.
