# Supplementary material for: The CHRNA5 rs16969968 variant is associated with MMP-9 expression and inflammatory signaling in COPD in a West Bengal population, India
Source: Front Pharmacol. 2026 Jun 24;17:1841945. doi: 10.3389/fphar.2026.1841945 (PMC13341563; doi:10.3389/fphar.2026.1841945)
Supplement: Supplementary file 1 [file Table1.docx]

| **Additive model** | | | | | | |
| --- | --- | --- | --- | --- | --- | --- |
| **Coefficients:** | **Estimate** | **Std. Error** | **z value** | **P value** | **Adjusted OR** | **95%CI** |
| Genotype | 0.87833 | 0.24875 | 3.531 | 0.000414 *** | 2.4069 | 1.4801-3.9437 |
| Age | 0.02453 | 0.01451 | 1.691 | 0.090847 | 1.0248 | 0.996-1.054 |
| Sex | 0.08729 | 0.30191 | 0.289 | 0.772491 | 1.091 | 0.603-1.98 |
| Smoking status | 17.07920 | 4.61134 | 3.704 | 0.000212 *** | 2.61*10^7^ | 2.17*10^4^-2.0885*10^12^ |
| Smoking duration | 0.30817 | 0.07880 | 3.911 | <0.001*** | 1.3609 | 1.1918-1.6370 |
| Cigarettes per Day | 1.29694 | 0.36290 | 3.574 | 0.000352 *** | 3.6581 | 2.0378-8.6556 |
| Pack years | 0.36302 | 0.16508 | 2.199 | 0.027869 * | 1.4377 | 1.0727-2.0878 |
| AIC: 299.7  Signif. codes: 0 ‘***’ 0.001 ‘**’ 0.01 ‘*’ 0.05 ‘.’ 0.1 ‘ ’ 1 | | | | |  |  |

| **Dominant Model** | | | | | | |
| --- | --- | --- | --- | --- | --- | --- |
| **Coefficients:** | **Estimate** | **Std. Error** | **z value** | **P value** | **Adjusted OR** | **95%CI** |
| Genotype | 0.90816 | 0.30280 | 2.999 | 0.002707 ** | 2.479 | 1.4097-4.4825 |
| Age | 0.02253 | 0.01439 | 1.566 | 0.117436 | 1.0228 | 0.9945-1.0520 |
| Sex | 0.11193 | 0.30076 | 0.372 | 0.709786 | 1.1185 | 0.6176-2.0328 |
| Smoking status | 17.42214 | 4.69274 | 3.713 | 0.000205 *** | 3.690^*^10^7^ | 2.8100*10^4^-4.8480*10^12^ |
| Smoking duration | 0.31326 | 0.07990 | 3.921 | <0.001*** | 1.678 | 1.1995-1.6414 |
| Cigarettes per Day | 1.31972 | 0.36798 | 3.586 | 0.000335 *** | 3.7424 | 2.1005-8.8716 |
| Pack years | 0.37166 | 0.16702 | 2.225 | 0.026061 * | 1.4502 | 1.0842-2.1103 |
| AIC: 303.14  Signif. codes: 0 ‘***’ 0.001 ‘**’ 0.01 ‘*’ 0.05 ‘.’ 0.1 ‘ ’ 1 | | | | |  |  |

| **Recessive Model** | | | | | | |
| --- | --- | --- | --- | --- | --- | --- |
| **Coefficients:** | **Estimate** | **Std. Error** | **Z value** | **P value** | **Adjusted OR** | **95%CI** |
| **Genotype** | 1.814511 | 0.638489 | 2.842 | 0.004485 ** | 6.1144 | 1.9772-22.6425 |
| **Age** | 0.027493 | 0.014472 | 1.900 | 0.057465 | 1.0282 | 0.9992-1.0591 |
| **Sex** | 0.006488 | 0.300665 | 0.022 | 0.982784 | 1.0065 | 0.5548-1.8224 |
| **Smoking status** | 17.087222 | 4.468648 | 3.824 | 0.000131 *** | 2.6418*10^7^ | 2.28*10^4^-1.7825*10^12^ |
| **Smoking duration** | 0.303604 | 0.076767 | 3.955 | <0.001*** | 1.3547 | 1.1890-1.6149 |
| **Cigarettes per Day** | 1.311590 | 0.349776 | 3.750 | 0.000177 *** | 3.712 | 2.05-87915 |
| **Pack years** | 0.345450 | 0.160926 | 2.147 | 0.031822 * | 1.4208 | 1.0582-2.0421 |
| AIC: 304.28  Signif. codes: 0 ‘***’ 0.001 ‘**’ 0.01 ‘*’ 0.05 ‘.’ 0.1 ‘ ’ 1 | | | | |  |  |

Supplementary Table 1: Multivariable logistic regression analysis.
