# Supplementary material for: The CHRNA5 rs16969968 variant is associated with MMP-9 expression and inflammatory signaling in COPD in a West Bengal population, India
Source: Front Pharmacol. 2026 Jun 24;17:1841945. doi: 10.3389/fphar.2026.1841945 (PMC13341563; doi:10.3389/fphar.2026.1841945)
Supplement: Supplementary file 2 [file Image1.pdf]

```
G1TTK0_RABIT      RRVFLHKLPKLLCMRSHADRYLAQEEEEEEEEEEEAGSGRRPRAPRNTLEAALESVRYIA   410
Q2MKA5_           RKIFLHKLPKLLCMRSHADRYFTQREEA--------EKDGGPK-SRNTLEAALDCIRYIT   403
P20420_RAT        RKIFLHKLPKLLCMRSHADRYFTQREEA--------ESGAGPK-SRNTLEAALDCIRYIT   388
A0A8I3RXC0_       RKIFLHKLPKVLCMRSHADRYFAQAGTG--------GA-GSPGAPRNTLEAALDSIRYIT   409
Q5IS51_Pan        RKIFLHTLPKLLCMRSHVDRYFTQKEET--------ESGSGPKSSRNTLEAALDSVRCIT   404
P30532_HUMAN      RKIFLHTLPKLLCMRSHVDRYFTQKEET--------ESGSGPKSSRNTLEAALDSIRYIT   404
G3QUJ5_           RKIFLHTLPKLLCMRSHVDRYFTQKEET--------ESGSGPKSSRNTLEAALDSIRYIT   404
A0A5F5PXF8_       RKIFLQKLPKLLCMRSHVDRYLTQKEET--------ECGSGPKSSRNTLEAALDSIRYIT   411
Q8SPU7_           RKIFLHKLPKLLCMRSHVDRYFSQKEEA--------RSSRGPRSSRNALEAALDSVRYIT   411
A0A287AQW1_PIG    RKIFLHKLPKLLCMQSHVDRYFGQKEET--------ESSSGPRSSRDTLEAALDSIRYIT   411
```

Supplementary Figure 1: Multiple Sequence Alignment of CHRNA5 protein sequence obtained from 10 mammalian species.
